# Chest examinations in children with real-time magnetic resonance imaging: first clinical experience

**DOI:** 10.1007/s00247-022-05421-8

**Published:** 2022-07-15

**Authors:** Franz Wolfgang Hirsch, Ina Sorge, Dirk Voit, Jens Frahm, Freerk Prenzel, Robin Wachowiak, Rebecca Anders, Christian Roth, Daniel Gräfe

**Affiliations:** 1 Department of Pediatric Radiology, University Hospital, Liebigstraße 20a, 04107 Leipzig, Germany; 2 Biomedical NMR, Max Planck Institute for Multidisciplinary Sciences, Göttingen, Germany; 3 Department of Pediatrics, University Hospital, Leipzig, Germany; 4 Department of Pediatric Surgery, University Hospital, Leipzig, Germany

**Keywords:** Children, Lungs, Magnetic resonance imaging, Preschool, Respiratory system, Thorax

## Abstract

**Background:**

Real-time magnetic resonance imaging (MRI) based on a fast low-angle shot technique 2.0 (FLASH 2.0) is highly effective against artifacts caused due to the bulk and pulmonary and cardiac motions of the patient. However, to date, there are no reports on the application of this innovative technique to pediatric lung MRI.

**Objective:**

This study aimed to identify the limits of resolution and image quality of real-time lung MRI in children and to assess the types and minimal size of lesions with these new sequences.

**Materials and methods:**

In this retrospective study, pathological lung findings in 87 children were classified into 6 subgroups, as detected on conventional MRI: metastases and tumors, consolidation, scars, hyperinflation, interstitial pathology and bronchiectasis. Subsequently, the findings were grouped according to size (4–6 mm, 7–9 mm and ≥ 10 mm) and evaluated for visual delineation of the findings (0 = not visible, 1 = hardly visible and 2 = well visualized).

**Results:**

Real-time MRI allows for diagnostic, artifact-free thorax images to be obtained, regardless of patient movements. The delineation of findings strongly correlates with the size of the pathology. Metastases, consolidation and scars were visible at 100% when larger than 9 mm. In the 7–9 mm subgroup, the visibility was 83% for metastases, 88% for consolidation and 100% for scars in T2/T1 weighting. Though often visible, smaller pathological lesions of 4–6 mm in size did not regularly meet the expected diagnostic confidence: The visibility of metastases was 18%, consolidation was 64% and scars was 71%. Diffuse interstitial lung changes and hyperinflation, known as “MR-minus pathologies,” were not accessible to real-time MRI.

**Conclusion:**

The method provides motion robust images of the lung and thorax. However, the lower sensitivity for small lung lesions is a major limitation for routine use of this technique. Currently, the method is adequate for diagnosing inflammatory lung diseases, atelectasis, effusions and lung scarring in children with irregular breathing patterns or bulk motion on sedation-free MRI. A medium-term goal is to improve the diagnostic accuracy of small nodules and interstitial lesions.

**Supplementary Information:**

The online version contains supplementary material available at 10.1007/s00247-022-05421-8.

## Introduction

### Why is real-time MRI for the thorax needed?

In young children, the quality of magnetic resonance imaging (MRI) of the lungs suffers primarily from motion artifacts caused by respiration and the moving heart. Nevertheless, in terms of examination frequency, lung imaging with MRI has prevailed over computed tomography (CT) in many pediatric radiology institutions.

Until now, the dogma has been that in young children who cannot hold their breath, data acquisition should be carried out during the low-motion expiratory phase. Various trigger techniques have been developed for this purpose. These include a breathing belt, a diaphragm navigator and a phase scout in liver tissue [1]. Recently, modern trigger techniques have also begun to use the periodicity of MR signals in a coil segment [2, 3].

Breath-triggered sequences, however, require very long examination times because the data are acquired only during a portion of the breathing cycle [4]. In addition, in about 10% of all lung MRI examinations, the result is still affected by artifacts due to children breathing irregularly or by bulk motion, leading to examinations not being diagnostically conclusive [5].

Another source of interference in lung MRI is artifacts caused by cardiac motion. These can be reduced by additional cardiac gating, radial readouts or averaging. However, all these methods are time-consuming [6, 7].

The entirely new approach to pediatric lung MRI described by this study abandons the prospective triggering and retrospective gating approaches. The basic idea is to speed up acquisition time for each image such that it is faster than any physiological motion. An examination free of motion artifacts is achieved at above 15 to 20 frames per s (50–66 ms) [8].

The recent introduction of a specific real-time MRI technique, fast low-angle shot technique 2.0 (FLASH 2.0), in two pediatric radiology departments in Germany has enabled such a high frame rate for daily routine evaluation [9] of moving organs such as the heart and joints, as well as for visualizing speech [10]. Of high clinical impact, this novel technique has recently been adopted to avoid motion artifacts in cranial MR examinations of non-sedated moving infants and young children [11]. Real-time MRI for this population has significantly reduced the need for anesthesia in cranial imaging [12].

These positive experiences in brain imaging encouraged employing real-time MRI techniques for pulmonary diagnostics in non-sedated infants and young children. This is because, especially in children unable to hold their breath, heart movement and movement of the child lead to limiting artifacts. The real-time method provides coverage of the whole lung in seconds in non-sedated free-breathing children while also allowing for bulk motion. The nearly 2 years of experience gained in using this technique for lung imaging has (for the first time) been reported here.

### Volume coverage sequences and MR equipment

The real-time MRI technique covered here was first described by Uecker and colleagues in 2010 and is commonly known as the fast low-angle shot technique 2.0 (FLASH 2.0) [13]. With rates of up to 50 frames per s, the organ or bulk movements of the child no longer cause relevant motion artifacts. Technical details of the FLASH 2.0 method have already been described in detail elsewhere [14].

**Fig. 1:**
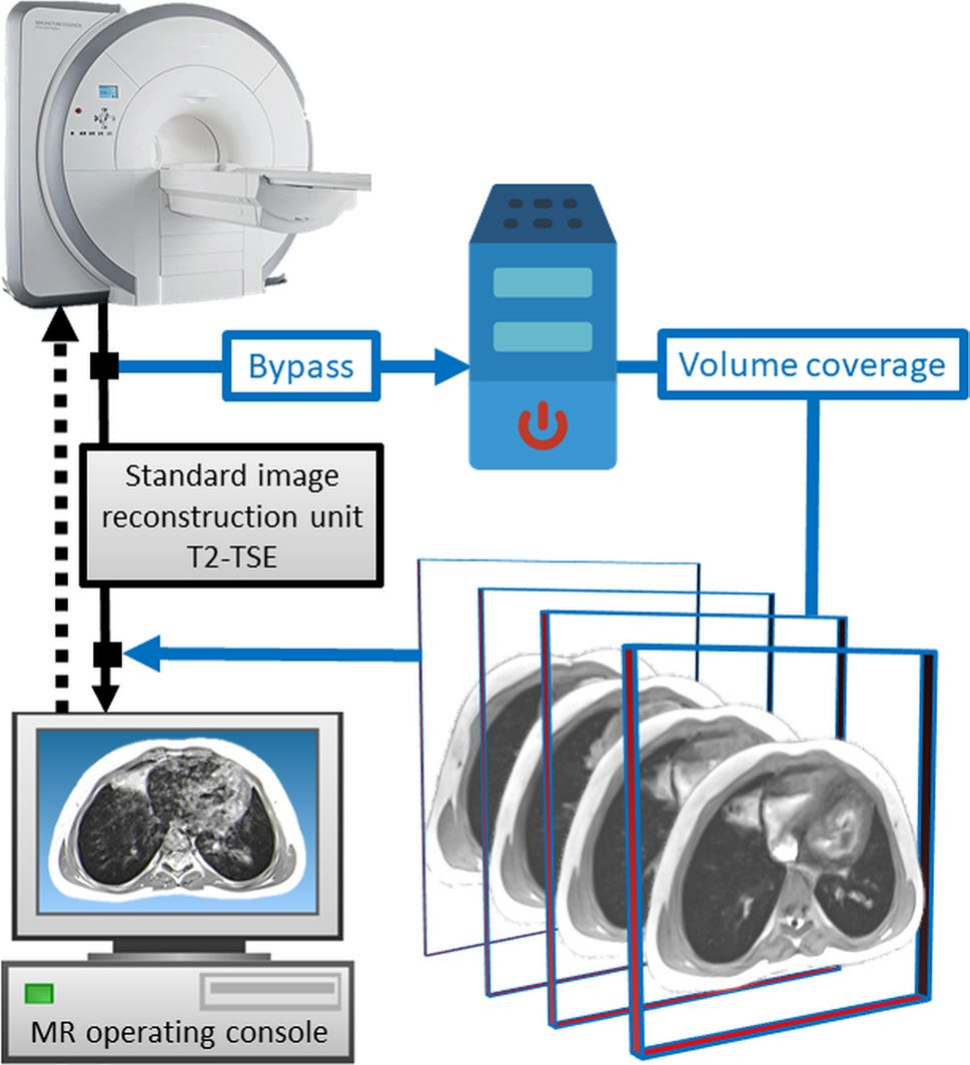
A block diagram of the connection of the dedicated image reconstruction unit with eight graphics boards (blue) for real-time magnetic resonance imaging (MRI). Also, the images of the volume coverage sequence as described here are generated in this bypass to the standard image data line of the MRI and then displayed at the MR operating console like any conventional sequence

Briefly explained, this unique MRI method is based on a highly undersampled radial gradient echo sequence. This is used in conjunction with a joint reconstruction of images and coil sensitivity maps charted by nonlinear inversion, with temporal regularization to the preceding frame. Processing is then done in a dedicated reconstruction unit with eight graphic processing units, and this computer is connected in bypass to a standard 3-T MRI system (Fig. 1). The real-time environment is currently adapted for Siemens 1.5- and 3-T MR scanners. In principle, however, it can be adapted to any MR system with appropriate programming, independent of the manufacturer. The licensor of the technology is the Max-Planck-Gesellschaft zur Förderung der Wissenschaften e.V./Germany. The sequences are integrated into the standard examination interface of the scanner, resulting in a completely regular planning sequence of the lung MR examination for the radiographer. The real-time pipeline is invisible to users, and the images are displayed with minimal temporal delay.

Initially, real-time MRI was tailored to visualize just a single layer of the body with high temporal resolution. A breakthrough evolution of this technique was the volume coverage sequence, which introduces a small slice shift with each image so that an entire organ volume can be scanned within a very short time [15]. Depending on the degree of overlap (60–90% of slice thickness) and thoracic dimensions, the time required to scan an entire organ is between 15 and 25 s. Children’s free-breathing, even with irregularities, is possible during the examination, as each image is intrinsically free of motion artifact.

#### Two sequence types for the lung and their tissue contrasts

**Fig. 2:**
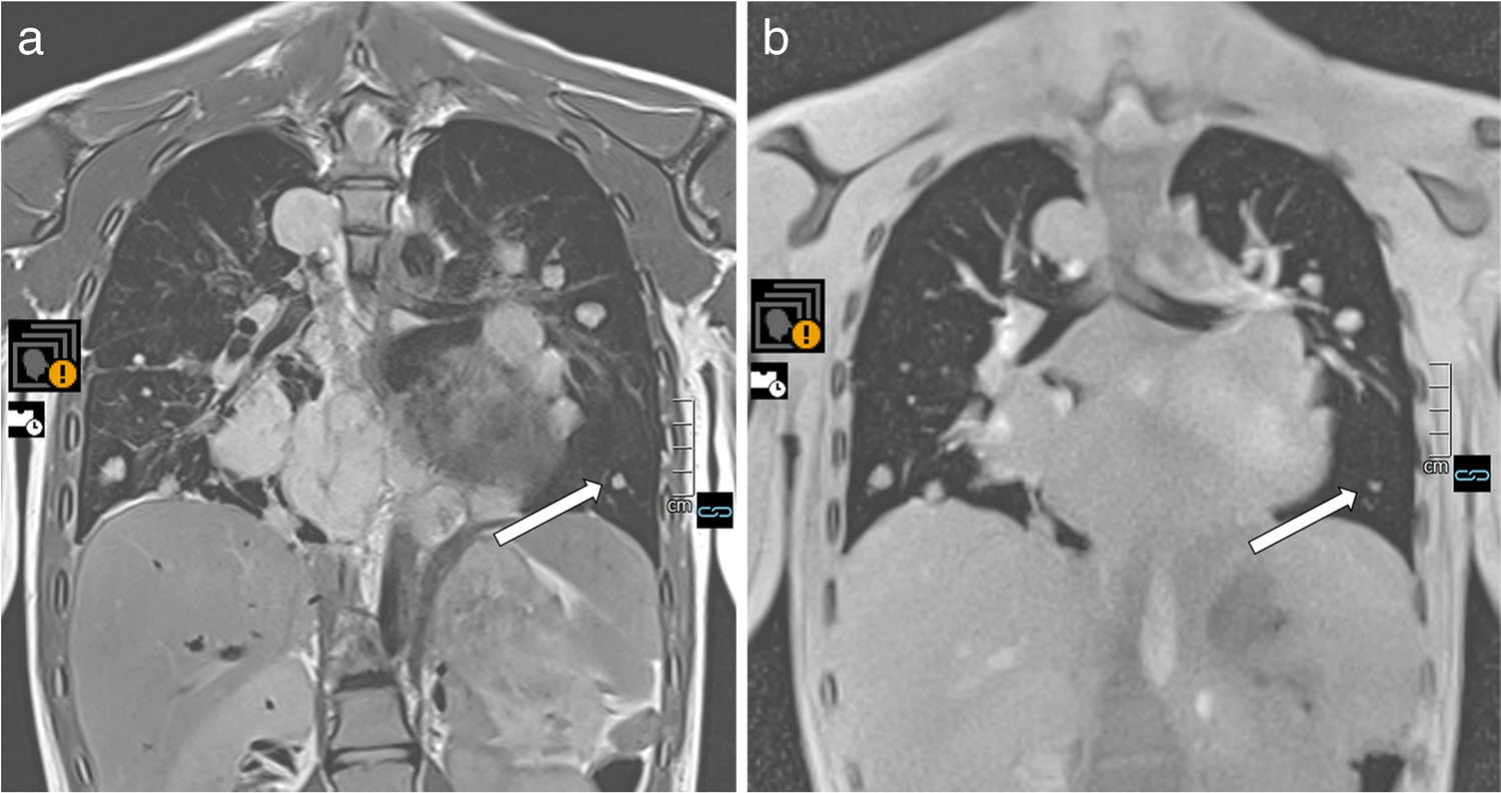
Contrast appearance of the proton-weighted volume coverage sequence (PDw-volume coverage) in a 16-year-old girl with lung metastases of various sizes from rhabdomyosarcoma of the right lower leg. Coronal images of lung metastases on T2 turbo spin-echo (TSE) (**a**) and real-time PDw-volume coverage (**b**) sequences. For patients with multiple lesions, the smallest identifiable metastasis was the lesion assessed. The smallest lesion in this girl measured 4 mm in diameter (*arrows*). Proton weighting did not allow further tissue differentiation, as the signal behavior of tissues and fluids is largely identical. The acquisition time for the triggered T2 TSE sequence was 4:55 min, the PDw-volume coverage acquisition time was 19 s in free-breathing (corresponding video: Online Supplementary Material 1)

FLASH 2.0 provides two major types of image contrast for lung diagnostics: a spin density contrast and a T2/T1 contrast. *The proton-weighted tissue contrast* provides images with a contrast impression of proton density–weighted (PDw) ultrashort echo time (UTE) sequences. The pulmonary vessels are very signal-rich due to the influx effect of gradient echo sequences. Although pathological lung findings usually contain many protons, they tend to be displayed with intermediate signal strength compared to the very signal-rich vessels. Due to a low flip angle, the energy absorption results in exceptionally low specific absorption rate values (Fig. 2 and corresponding video: Online Supplementary Material 1).

**Fig. 3:**
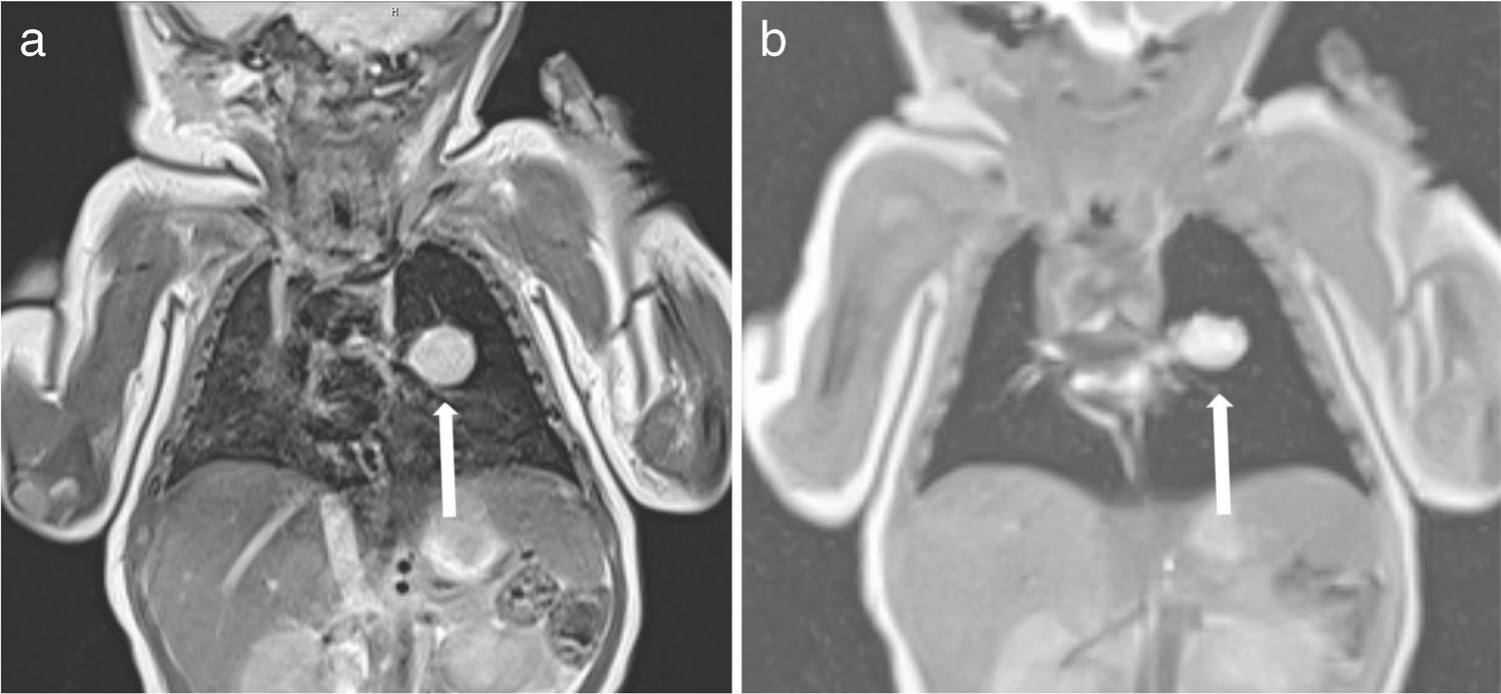
Contrast appearance of the T2-weighted volume coverage sequence in a 2-day-old girl with a bronchocele *(arrows*) of the left upper lobe bronchus and hyperinflation of the entire left upper lobe in coronal projection. The hyperinflation of the upper lobe was recognizable only to a limited extent on the T2 sequences. **a** An image of the bronchocele on the T2-turbo spin-echo (TSE) standard sequence. **b** An image of the real-time T2-volume coverage sequence. The acquisition time of the triggered T2 TSE sequence was 3:50 min; the T2-volume coverage acquisition time was 12 s in free breathing “(corresponding video: Online Supplementary Material 2)

An additional refocusing gradient was applied to create a *T2/T1 contrast* with a FLASH 2.0 sequence. This T2/T1 weighting would, in principle, be advantageous for the detectability of lung lesions and further tissue differentiation (Fig. 3 and corresponding video: Online Supplementary Material 2). However, this sequence scheme requires a period of motion quiescence to produce a stable steady-state and thus the desired T2-type contrast (approximately 1–2 s). Although motion blurs do not occur during rapid lung movements, in some cases the signal intensity of a lesion changes depending on the speed of movement of the diaphragms (Fig. 3 and corresponding video: Online Supplementary Material 2).

This study aimed to compare the two new real-time volume coverage sequences retrospectively with standard chest MRI examinations performed for medical reasons regarding the delineability of pathological lesions.

## Materials and methods

This retrospective study was approved by the local ethics committee (University Hospital Leipzig; 228/20-ek). Written informed consent was obtained from the patients or their legal guardians.

The study included children ages 0 to 16 years who underwent lung MRI for medical reasons. Patients were selected consecutively with all patients referred for a lung MRI also receiving an examination with the real-time technique. The recruitment of patients took place from Feb. 1, 2020, to Jan. 31, 2022. All patients without pathological findings on standard MRI were excluded from further analysis. The standard lung protocol consisted of two T2 turbo spin echo (TSE) sequences with respiratory triggering in axial and coronal planes and one T2-TSE sequence with fat saturation in the axial plane (Online Supplementary Material 3). In 30 of the 87 participants (35%), a self-gated PDw–UTE sequence was also conducted [16]. All examinations were performed on a 3-T scanner (Prisma Fit; Siemens, Erlangen, Germany). The real-time volume coverage sequences were subsequently obtained. We compared the two new volume coverage sequences with the overall result of our standard MRI protocol (plus a UTE sequence, if available). PD- and T2/T1-weighted volume coverage sequences were acquired in coronal and axial planes (Table 1). The duration for these four sequences was 60–80 s, depending on thoracic size. Data acquisition was carried out during free, spontaneous breathing (Table 1).

**Table 1:** Acquisition parameters for an axial thoracic volume coverage sequence (in a 2-year-old child)

	PDw-VC	T2/T1-VC
Contrast	PD	T2/T1
Field of view (mm^2^)	300 × 300	300 × 300
Image matrix	256 × 256	256 × 256
Resolution (mm^2^)	1.2 × 1.2	1.2 × 1.2
Thickness (mm)	3	3
Section shift (mm)	0.45 (15%)	0.45 (15%)
TR/TE (ms)	3.19 / 2.07	3.82 / 1.91
Spokes per section	31	31
Flip angle/degree	6	30
Time per section (ms)	99	118
Number of sections	180	180
Time per volume (s)	18	18
Motion compensation	No trigger Free breathing	No trigger Free breathing

*PD* proton density contrast, *PDw-VC* proton density weighted volume coverage sequence, *T2/T1* T2/T1 contrast, *T2/T1-VC* T2/T1 weighted volume coverage sequence, *TE* echo time, *TR* repetition time

To assess the diagnostic reliability of real-time MRI, lesions detected by standard MRI were grouped into six categories: metastases and tumors, consolidation, scars, hyperinflation, interstitial lung disease and bronchial ectasia. Lesions were further subcategorized into three groups by size: group 1 (4–6 mm), group 2 (7–9 mm) and group 3 (≥ 10 mm). In the case of irregular lesions, we recorded the largest short axis measurement. Scars were defined as linear, local strand-like lesions, mostly after lung surgery and after inflammation. Finally, a pediatric radiologist with 23 years of experience in pediatric lung MRI (F.W.H.) determined whether each lesion on the standard MRI was also reliably detected on the new volume coverage sequences, using a three-level Likert scale. Conspicuity of lesions was classified as follows: 2, clear detection of the lesion was possible; 1, the lesion was detectable only in the knowledge of its presence on the standard MRI, and 0, the lesion was not visible at all. Only a score of 2 was considered diagnostically sufficient. This evaluation was performed separately for the PDw-volume coverage and T2-volume coverage images, combined for both coronal and axial orientations.

Statistical tests on group differences were deliberately omitted because of the small size of the subgroups. Nevertheless, it was decided to report the results and potential of the first clinical experience of this technique in the pediatric population.

## Results

A total of 169 lung MRI examinations with volume coverage sequences were performed. Indications for a lung MRI can be divided into 5 groups: oncological staging or follow-up (102), cystic fibrosis follow-up (29), suspected inflammatory lung disease (20), suspected malformations of the lung (14) and suspected interstitial lung disease (4). Eighty-two examinations revealed normal findings on conventional lung MRI; these were excluded from further assessment. Ultimately, 87 children between 0 and 16 years of age (median age: 6.5 years; 41 girls) with a pathological lung MRI finding were identified and included in the comparative analysis. For each lung MRI, only the primary lesion was assessed. If several lesions were present, only the smallest lesion was evaluated to avoid bias. For instance, in a patient with 30 lung metastases, only 1 lesion (the smallest visible) was evaluated.

**Fig. 4:**
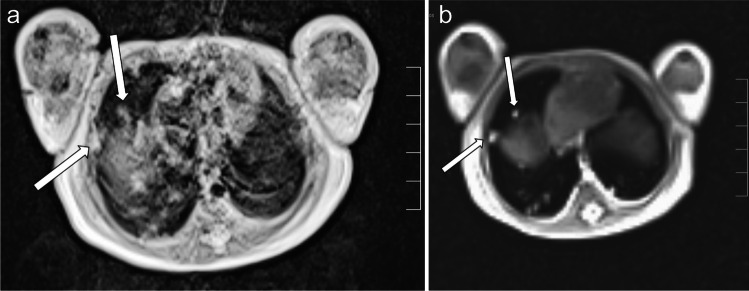
A 3-day-old boy with pneumonic nodules (*arrows*) due to neonatal pneumonia in axial projection. **a** A T2-turbo spin echo image with respiratory artifact during uneven breathing lasted 4:05 min. **b** A T2-volume coverage image without respiratory artifacts lasted only 14 s in free breathing. The detectable size of nodules in this neonate was 3–4 mm (corresponding video: Online Supplementary Material 4)

**Fig. 5:**
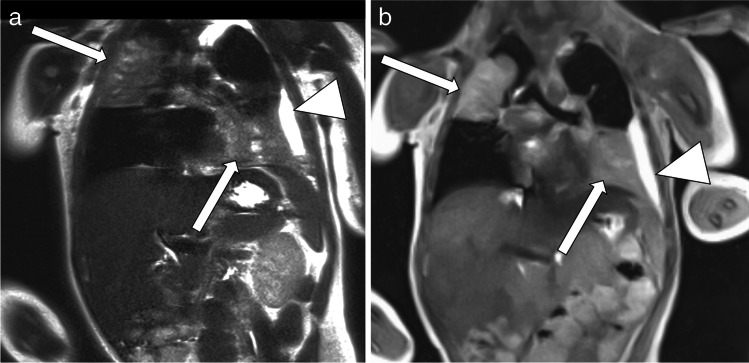
A 2-year-old girl diagnosed with bilateral pneumonia (*arrows*) with abscesses due to staphylococcus and left pleural effusion (*triangle*) in coronal projection. **a** A T2-turbo spin echo sequence lasted 3:30 min. **b** A T2-volume coverage sequence lasted 16 s in free breathing (corresponding video: Online Supplementary Material 5)

**Fig. 6:**
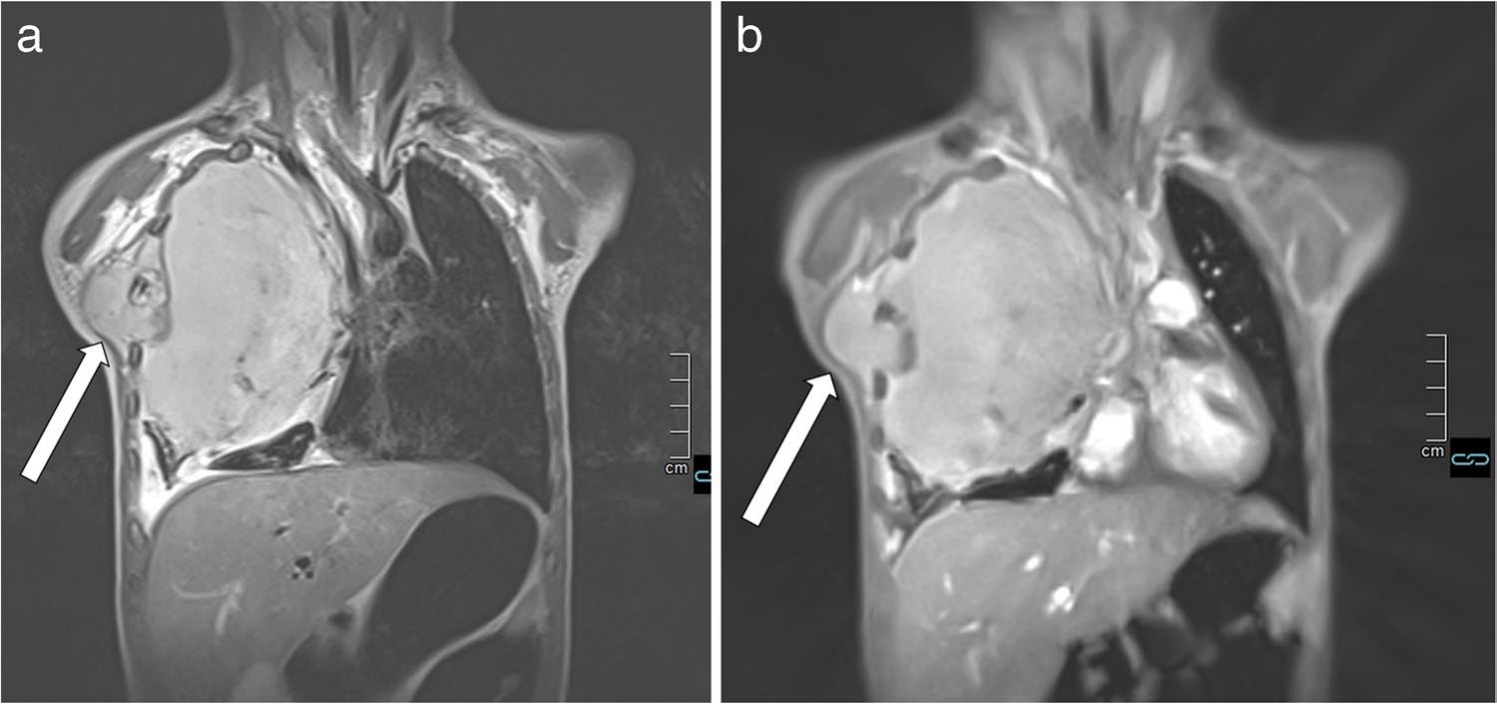
A 3-and-a-half-year-old boy with Ewing sarcoma of the right 5th rib (*arrows*) in coronal projection. **a** A T2-turbo spin echo sequence lasted 3:20 min. **b** A T2-volume coverage sequence lasted 19 s in free breathing. In such voluminous tumors of the thoracic wall and mediastinum, the T2-volume coverage sequence shows consistently good results because there is no fluctuating signal intensity due to the immobility of the tumor (corresponding video: Online Supplementary Material 6)

The real-time volume coverage acquisition with both coronal and axial projections took between 26 and 50 s, depending on the number of slices needed to cover the whole chest. In comparison, acquisition time for T2-TSE sequences was between 7 and 12 min, depending on the size of the thorax and the efficiency of respiratory triggering. The following three video examples are intended to give the reader a visual impression of the T2-volume coverage image and thus of the image quality that can currently be achieved (Fig. 4 and corresponding video: Online Supplementary Material 4) (Fig. 5 and corresponding video: Online Supplementary Material 5) (Fig. 6 and corresponding video: Online Supplementary Material 6).

Though not all 87 lesions could be identified on real-time MRI with volume coverage sequences, the visibility of the lesions in volume coverage sequences correlated strongly with the size of the findings. The smallest lesion that was readily recognizable had a diameter of 4 mm.

**Fig. 7:**
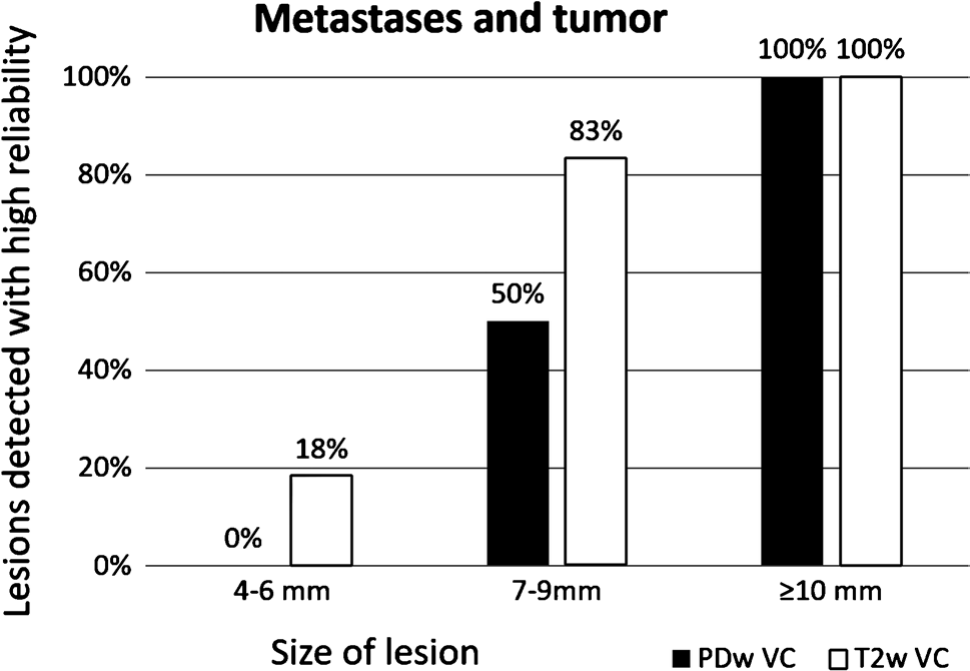
The delineation of metastases and tumors on the two volume coverage (VC) sequences depending on lesion size. *PDw-VC* proton density weighted volume coverage

Metastases and tumors (*n* = 24) could be seen in size group 1 (4–6 mm) in up to 18% of the studies. In size group 2 (7 to 9 mm), between 50% (PDw-volume coverage) to 83% (T2-volume coverage) of lesions were detected. In size group 3 (≥ 10 mm), all metastases and tumors were reliably detected (100%) in both weightings (Fig. 7).

**Fig. 8:**
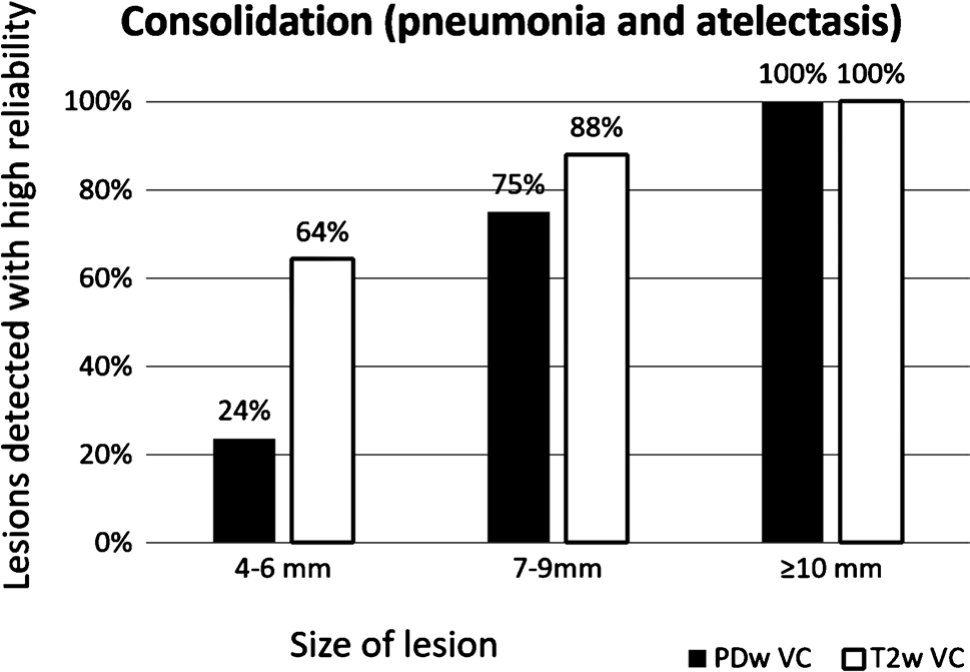
Detectability of consolidation of the lung (pneumonia or atelectasis) on the proton-weighted volume coverage (PDw-VC) and T2-VC sequences depending on lesion size

In detecting consolidation, such as pneumonic infiltrates and atelectasis (*n* = 35), the T2-volume coverage sequence tended to be more sensitive than PDw-volume coverage. In size group 1 (4–6 mm), 64% of the findings were seen in the T2-volume coverage group. In group 2 (7–9 mm), 88% were seen, and in group 3 (≥ 10 mm), all consolidations were detected (Fig. 8).

**Fig. 9:**
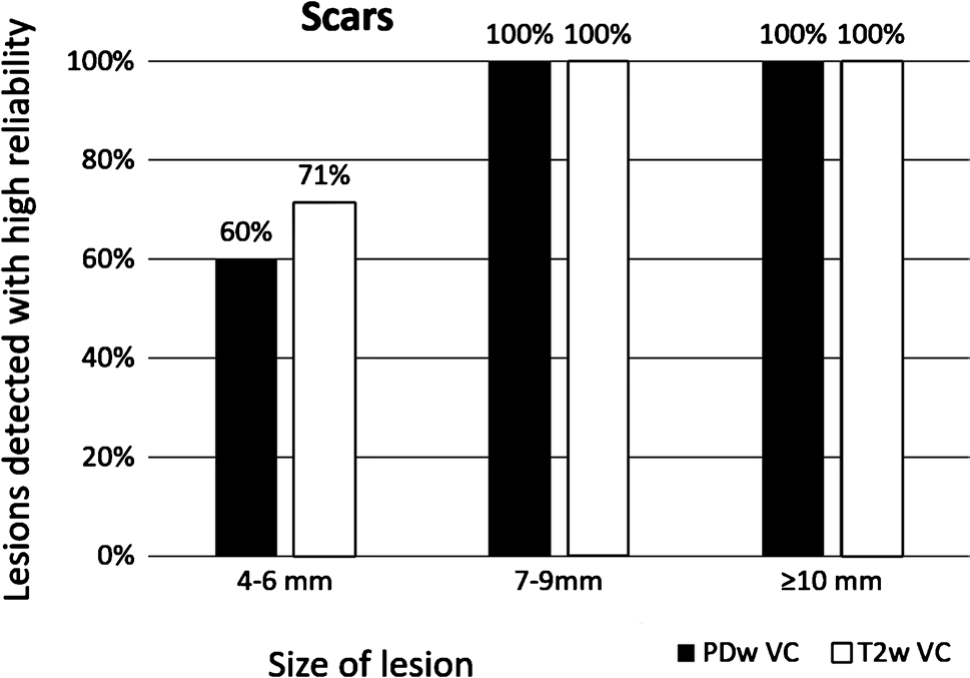
Detectability of scarring (postoperative or post-inflammatory) on the proton-weighted volume coverage (PDw-VC) and T2-VC sequences depending on lesion size

Scarring (*n* = 12) could be reliably identified even with small diameters (postoperative or post-inflammatory). They were readily detectable in both weightings of the volume coverage sequences, even at small sizes: The visibility was 60–71% for group 1 and 100% in size group 2 (7–9 mm) (Fig. 9).

Volume coverage sequences failed to detect bronchiectasis (*n* = 4), hyperinflation (*n* = 3) and interstitial lung changes (*n* = 9) reliably, each rating below 2. However, these findings are referred to in the literature as MR-minus pathology, hardly visible even on standard MRIs.

## Discussion

Our results show that real-time MRI is feasible even in small children and provides images of the lung free of motion artifact. It is already adequate for diagnosis and follow-up examinations of inflammatory lung diseases, atelectasis, pleural effusions and lung scarring in non-sedated children with bulk motion or with irregular breathing patterns. The acquisition time is only 6–7% of a standard respiratory-triggered TSE sequence though this time saving is not crucial for this novel concept. In fact, the focus of real-time MRI in this new approach in lung imaging is to avoid motion artifact in free-breathing small children without sedation. However, the method still has significant limitations when compared to a standard lung MRI.

The hurdles of diagnostic use of real-time MRI for lung imaging are expectedly high. This is because the sequences were originally optimized for cardiac examinations in adults. The gradient echo sequences initially used had proton-weighted contrast [13], whereas T2 contrasts have been predominantly preferred for pediatric lung imaging [17, 18].

Of the two volume coverage sequences tested, the T2-volume coverage sequence was superior in detecting pathological findings. However, it suffers from fluctuations in signal intensity with fast-moving structures. This can result in a lower signal of smaller pathological findings (e.g., smaller lung metastases), which may then escape detection.

However, if the pathological finding is not within the lung but in the mediastinum or thoracic wall, hardly any signal fluctuation in the T2-volume coverage sequence is present during breathing [19], which supports the generation of the steady state in these gradient echo sequences. The T2-volume coverage images also showed occasional veil-like artifacts in the coronal orientation, which did not appear in the same way in PDw-volume coverage. The origins of these have not yet been clarified. Nevertheless, further development and optimization of this T2/T1 contrast-type volume coverage sequence seems worthwhile.

Compared to T2-volume coverage, the PDw-volume coverage sequence showed very little variation in signal intensity during breathing. However, it shows only low tissue contrast differences compared to T2-/T1-weighted sequences [20]. This, for example, renders differentiation of effusion and consolidation very difficult. In our patients, smaller findings, clearly recognizable in the T2-volume coverage sequence, were often missed in PDw-volume coverage (Fig. 4). This is probably also caused by the low signal contrast between the normal lungs and the pathological lesions. MR-minus pathology, such as interstitial processes, cysts and hyperinflation, cannot be reliably detected with real-time MRI regardless of the weighting. However, this is an inherent problem of MRI diagnostics in lung imaging not specific to real-time MRI [21].

Another interesting secondary aspect arises from the high blood signal in the pulmonary vessels, which often dominates the impression in volume coverage sequences. This bright blood signal is typical for many gradient echo sequences [22]. While, on the one hand, the bright vessels distract from small, low-signal findings within the lungs, the gradient echo sequences may, on the other hand, evolve in the future with some modifications such as non-contrast, free-breathing angiography. For example, local vascular rarification, as in congenital bronchus atresia with local hyperinflation or Swyer-James syndrome, may be detectable with this technique.

Further optimization of the volume coverage sequences presented in this report is being tested. This optimization is primarily aimed at increasing the matrix and optimizing the number of spokes. The use of 17 spokes, instead of 31 at present, seems to be sufficient.

To further reduce the variability of the contrasts in the T2/T1 weighting, the section shift of 15% could be reduced even further, thus allowing measurements in the same position for a longer time. Lastly, the interfering flow signals in the vessels can also be eliminated by partial saturation [23] or bilateral co-running saturation volumes, which is foreseen in the latest version of the sequences.

The limitation of the method in its present form is the unreliable detection of small findings in the 4–6 mm size group, also caused by the inconsistency of signal intensity in the T2-type volume coverage sequence. The visualization of the MR-minus pathology, including hyperinflation, cysts and interstitial parenchymal changes, is a limitation. However, it is difficult in standard lung MRIs, too, and thus is not a disadvantage specific to real-time MRI [24]. As a further limitation to this study, we did not assess the hilar lymph nodes as a separate category. The reason was that our focus was on assessing the moving lung. At this point in time, we would prefer assessing mediastinal lymph nodes with a standard MRI. This was a retrospective study. It is difficult to compare different sequences. This is particularly true for lung MRI, as there is no gold standard in lung imaging. Additionally, the extent of bulk motion in children has not been systematically investigated, as macro movements cannot be systematically classified. Therefore, we only report our experience that the irregular breathing and small macro movements of the children did not lead to any relevant motion artifacts. In summary, it can be stated that for specific indications such as pneumonia or atelectasis and the detection of scars in the lungs, real-time MRI has proven to be well suited.

The new concept of real-time MRI of the lung is not fundamentally challenged by the current limitations. But it requires further development so that real-time lung MRI becomes even more sensitive with respect to small lesions in the future. The aim of the method should be sensitive imaging of lung pathology without artifacts. It may not be possible to achieve identical information like from a conventional state-of-the-art MRI lung examination, but specific indications can be conclusively clarified with this simple, fast and artifact-free technique. Further research is needed on these indications. Perhaps it will be possible to combine the advantages of UTE sequences, which generate a high signal of the lung structures [25], with the speed and artifact-free nature of the real-time MRI to create a diagnostic win-win situation.

## Conclusion

After evaluating 87 lung MR examinations with pathological findings in the chest wall and the lungs included, it has been proven that real-time examination is a significant innovation for pediatric radiology regarding motion-artifact-free lung images. This first publication of preliminary results in children and description of the current limitations could possibly encourage other groups to refine this innovative technique.

## Supplementary Information

Below is the link to the electronic supplementary material.

Supplementary file1 (MP4 12.3 MB)

Supplementary file1 (MP4 7.29 MB)

ESM 3 (DOCX 14.7 KB)

Supplementary file1 (MP4 4.90 MB)

Supplementary file1 (MP4 8.11 MB)

Supplementary file1 (MP4 6.35 MB)
