# Supplementary material for: Chest examinations in children with real-time magnetic resonance imaging: first clinical experience
Source: Pediatr Radiol. 2022 Jul 15;53(1):12–20. doi: 10.1007/s00247-022-05421-8 (PMC9816257; doi:10.1007/s00247-022-05421-8)
Supplement: Supplementary file 3 — (DOCX 14.7 KB) [file 247_2022_5421_MOESM3_ESM.docx]

**Supplemental Table 1** Scan parameters for the standard thoracic sequences employed in this study

| **Parameter** | **T2-TSE** | **T2-TSE with fat suppression** | **PDw–3D UTE** |
| --- | --- | --- | --- |
| **Repetition time (ms)** | 1000–2500 | 1000–2500 | 3.1 |
| **Echo time (ms)** | 56 | 57 | 0.05 |
| **Flip angle** | 140° | 140° | 5° |
| **Signal averaging** | 1 | 1 | 1 |
| **Slice orientation** | Axial and coronal | Axial | Coronal and MPR reconstructions |
| **Field of view (mm)** | 350 x 230 | 350 x 230 | 465 x 465 |
| **Phase resolution** | 70% | 70% | 100% |
| **Matrix** | 384 × 204 | 384 × 204 | 320 × 320 |
| **Voxel size (mm)** | 1.1×0.9 ×3.0 | 1.1×0.9×3.0 | 1.5×1.5×1.5 |
| **Parallel acquisition technique (PAT)** | PAT 2 | PAT 2 | Not applicable |
| **Contrast medium** | Not applicable | Not applicable | No |
| **Acquisition time (min)** | 3-8 | 3–8 | 6–8 |
| **Motion compensation** | Triggered with phase scout in liver | Triggered with phase scout in liver | Self-gated |

*MPR* multiplanar reformation, *PDw-3D UTE* Proton density weighted 3D ultrashort echo time, *T2-TSE* T2 turbo spin echo
